# Case report: A clinical report of photodynamic neoadjuvant combined with fluorescent laparoscopic localization robotic surgery for the treatment of patients with advanced colorectal cancer combined with obstruction

**DOI:** 10.3389/fimmu.2024.1403613

**Published:** 2024-09-04

**Authors:** Xiaodong Huang, Xiaowen Han, Weidong Li, Jiayi Zhang, Bin Ma, Yuhan Wang, Zhenyu Yin, Lei Gao, Jianming Shi, Ewetse Paul Maswikiti, Hao Chen

**Affiliations:** ^1^ The Second Hospital and Clinical Medical School, Lanzhou University, Lanzhou, China; ^2^ Key Laboratory of the Digestive System Tumors of Gansu Province, The Second Hospital and Clinical Medical School, Lanzhou, China; ^3^ Department of Surgical Oncology, Lanzhou University Second Hospital, Lanzhou, China

**Keywords:** colon cancer, case report, photodynamic, neoadjuvant therapy, fluorescence laparoscopy

## Abstract

The incidence of colorectal cancer is relatively high in our country, with the majority of patients being diagnosed at an advanced stage. For individuals with advanced-stage colorectal cancer, conversion or neoadjuvant therapy is frequently necessitated to facilitate surgical intervention and achieve a curative effect. And about 10% to 30% of colon cancer patients are complicated with intestinal obstruction. Surgical intervention remains the primary treatment for managing intestinal obstructions, albeit with a considerable risk of perioperative mortality and an increased likelihood of postoperative complications. PDT, as a neoadjuvant treatment for colon cancer, can shrink the local tumor and relieve obstruction, and is effective in colon cancer combined with obstruction. Robotic surgery has the advantages of high stability and low trauma, and compared with laparoscopic colon cancer surgery, robotic surgery can achieve better results. Fluorescent laparoscopic clarifies the location and size of the tumor lesion, allowing for greater precision when removing colon cancer lesions in robotic surgery. Therefore, in the treatment of colon cancer, PDT can offer an opportunity for surgery after relieving obstruction in patients with obstructive colon cancer. Additionally, when combined with fluorescent laparoscopic robotic colon cancer surgery, it provides a novel treatment approach for patients with obstructive colon cancer. Preoperative photodynamic neoadjuvant therapy combined with robotic colon cancer surgery has not yet been reported. Here, we report a case of colon cancer with obstruction, preoperative TNM stage was T4N1, and the lesion had caused intestinal stenosis. After four sessions of PDT, the patient’s intestinal lumen was unobstructed and the lesion had regressed. After evaluation, fluorescent laparoscopic localization and visualization of lymph nodes combined with robotic colon cancer resection were performed. Postoperative pathology showed that the patient’s tumor regression grade was grade 1. The patient’s tumor was completely resected with good resection effect. No tumor invasion was found on both sides of the resection margin, and the patient did not relapse after surgery.

## Introduction

Colorectal cancer exhibits a high incidence and mortality rate in China, The 2020 Global Cancer Statistics data shows that it ranks second in incidence and fifth in mortality among all types of cancer (1). Approximately 10% to 30% of colon cancer patients suffer from intestinal obstruction. Given the smaller lumen of the descendants colon compared to the ascendants colon, feces in the descendants colon tend to be more solid, and tumors often exhibit infiltrative growth, resulting in obstructive cases occurring predominantly in the descendants colon, accounting for 70% of instances. In contrast, such cases in the ascendants colon makeup 20% to 30% (2). Currently, the primary modality for managing colonic cancer with obstruction remains surgical intervention. This can adopt a one-stage colostomy, followed by tumor resection in the second stage and subsequent anastomosis in the third stage. Alternatively, a one-stage tumor resection with simultaneous colostomy and subsequent anastomosis may be performed. Another option is to pursue a one-stage tumor resection with simultaneous anastomosis. However, all three surgical approaches have their limitations and fall short of achieving optimal outcomes (**Supplementary Table 1**) (3–10). PDT uses a light to irradiate the tumor site enriched with photosensitizers, then the interactions between light, photosensitizers, and oxygen occur to trigger a photochemical reaction that produces ROS, ultimately leading to destruction of tumor cells (11). Photodynamic neoadjuvant therapy enables the reduction of local tumor size, alleviate intestinal obstruction, transform emergency surgery for obstructive colon cancer into elective surgery, enhance the resection rate in robotic surgery, improve anal retention rates for patients, minimize postoperative complications and recurrence rates. Ultimately, this treatment approach extends patient survival time and enhances their quality of life while optimizing surgical resection outcomes (12). PDT offers several advantages, including minimal invasiveness, high targeting precision, and excellent efficacy in patients with obstructive colon cancer. Consequently, PDT holds an indispensable advantage in the treatment of such patients. Currently, surgical intervention remains the sole curative approach for colon cancer. In this regard, robotic surgery demonstrates safety and feasibility compared to laparoscopic surgery (**Supplementary Table 2**) (13–15). Fluorescence laparoscopy enables precise localization of tumor lesions, providing accurate information on their position and size. Consequently, the combination of fluorescence-guided robotic-assisted colon cancer resection surgery can significantly enhance the precision of resection boundaries, thereby reducing postoperative complications and minimizing recurrence rates for patients. In this report, we present a compelling case demonstrating the successful alleviation of colon cancer obstruction through the synergistic application of PDT and fluorescence-guided laparoscopic robot-assisted surgery. The patient exhibited remarkable tumor resection outcomes and achieved a favorable postoperative recovery, thereby affirming the safety and feasibility of employing PDT in conjunction with fluorescence-guided laparoscopic robot-assisted surgery for colon cancer treatment. These findings offer novel insights and directions for enhancing clinical management strategies against colon cancer.

## Clinical data

**Figure 1:**
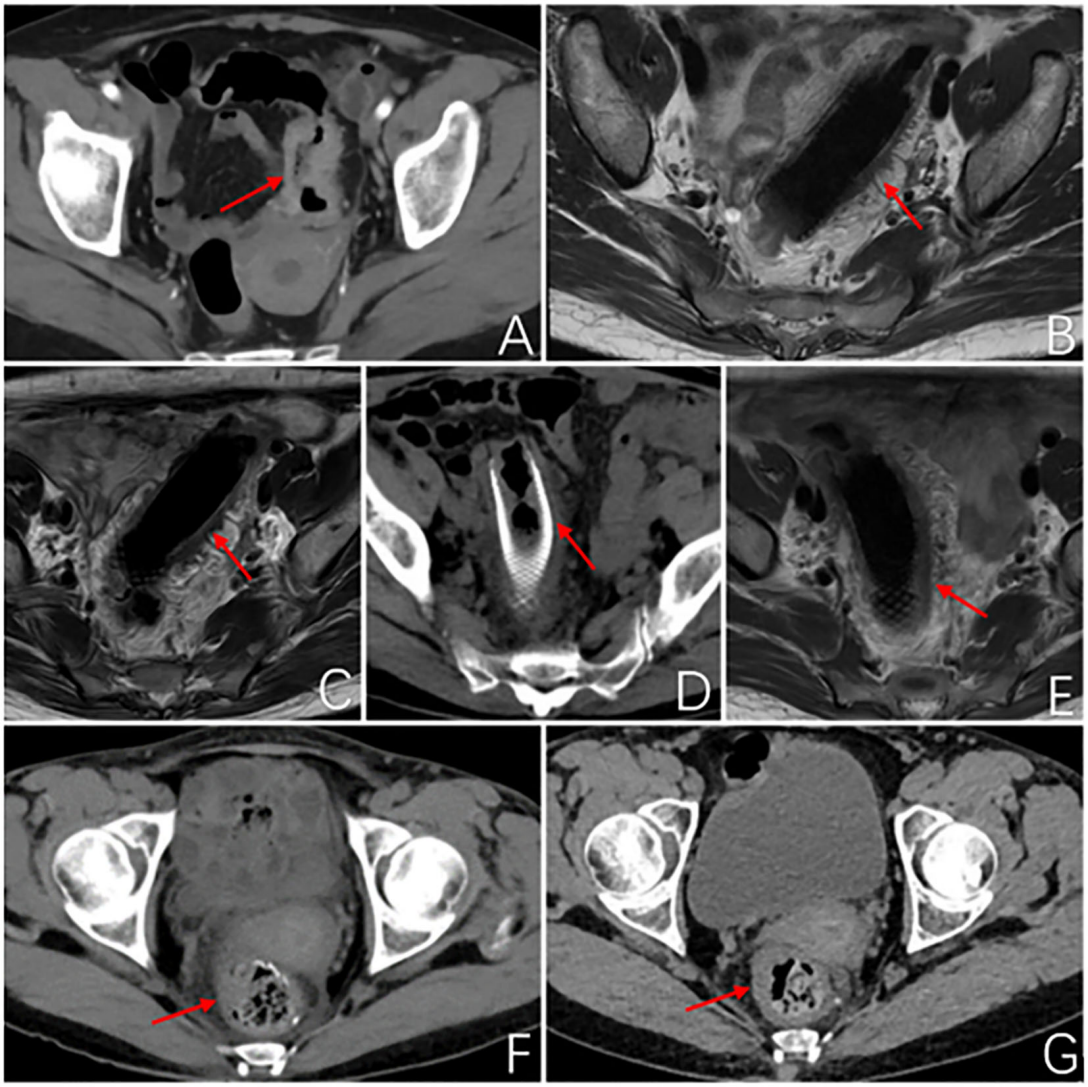
Imageological examination. **(A)** Abdominal enhanced CT at first admission: The local wall of the sigmoid colon shows circumferential thickening with mild enhancement, resulting in a narrow lumen and roughness around the wall. Consider sigmoid colon cancer, preliminary staging T4N1. **(B)** MR rectal scan after 3 months of PDT: The image shows a metallic internal fixator within the lumen of the sigmoid colon, with corresponding mild thickening of the intestinal wall. The proximal and distal ends of the stent exhibit uneven thickening of the intestinal wall that protrudes into the lumen. **(C)** MR rectal scan 4.5 months after PDT: Within the lumen of the sigmoid colon, a metallic internal fixator shadow is visible. Mild thickening of the bowel wall where the stent is located, uneven thickening of the bowel wall proximal and distal to the stent and protruding into the lumen, proximal luminal stenosis slightly better than before. **(D)** Abdominal CT 6 months after photodynamic surgery: After colonic stent placement, thickening of the wall and grossness of the tube is seen. **(E)** MR Rectal plain scan 6 months after photodynamic operation: Changes after colonic stent placement, uneven thickening of the proximal and distal bowel wall of the stent and protruding into the lumen, this time the proximal and distal bowel wall of the stent thickened to a lesser extent. **(F)** Abdominal CT 2 months after robotic surgery: No obvious abnormal thickening of the anastomotic wall after radical surgery for colon cancer. **(G)** Abdominal CT 5 months after robotic surgery: After radical resection of colon cancer, the anastomotic wall does not thicken. The red arrows demonstrate the site of the tumor lesion, the location of the metal stent, and the postoperative anastomosis.

**Figure 2:**
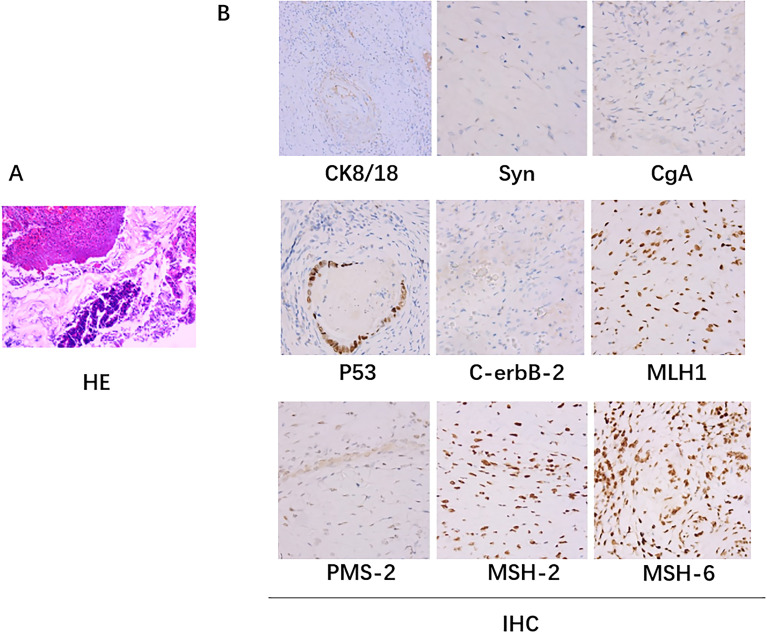
Pathologic biopsy results. **(A)** The hematoxylin-eosin (H&E) staining: A small amount of residual well-differentiated adenocarcinoma component in the ulcerative lesion area, with cancer invasion into the subserosal layer (pT3), without definite involvement of blood vessels or nerves; tumor regression grade (TRG): Grade 1 (single or small foci of residual cells); no cancer invasion in the resected margins on both sides. **(B)** Immunohistochemical staining: there was no lymph node metastasis (0/24); immunohistochemistry staining results: cancer cells CK8/18+,Syn-, CgA-, p53 (+), C-erbB-2 (0), Ki67 (40%+); MLH1+, PMS-2+, MSH-2+, MSH-6+.

The patient, female, 51 years old, was admitted to our hospital on December 6, 2022 for “difficulty in defecation for more than 3 months”. The enhanced Computed Tomography (CT) examination of abdomen showed: sigmoid colon cancer, preliminary stage T4N1 (**Figure 1**). Enteroscopy showed: the colonoscope reached a depth of 20cm and revealed an elevated lesion causing luminal constriction, impeding further advancement of the scope. The lesion exhibited a firm texture with a constrictive growth pattern and surface ulceration (**Supplementary Figure 1**). The pathological examination results revealed the presence of moderately differentiated adenocarcinoma in the colon (**Figure 2**). Immunohistochemical staining results showed that the cancer cells expressed CK8/18(+), p53 (mutant), Syn(-), and had high expression of Ki-67 (70%+).Genetic testing results: K-RAS gene: wild type; N-RAS gene: wild type; B-Raf gene: wild type. After multidisciplinary discussion, it was recommended that the patient undergo PDT as an effective intervention for alleviating the existing obstruction.

**Figure 3:**
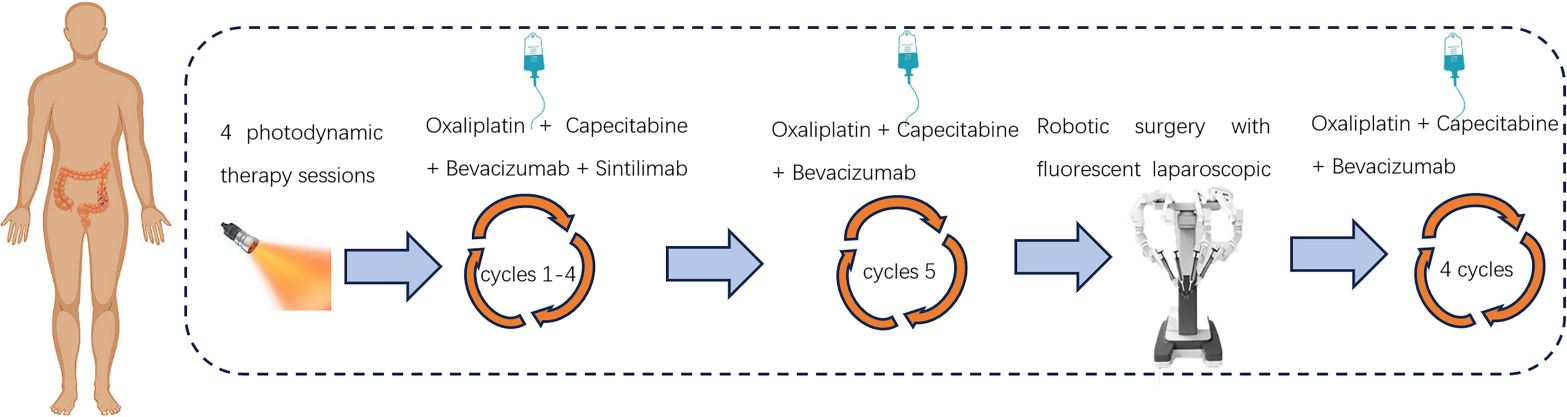
Treatment flow chart.

After a negative skin test for hematoporphyrin injection prior to photodynamic surgery, the patient was injected with the domestic photosensitizer Hematoporphyrin Injection (25 mg, Chongqing Meile Biopharmaceutical Co., Ltd.). The injection administered an intravenous dose of 3 mg/kg along with 250 ml of normal saline over approximately 60 minutes, ensuring protection from light exposure. Following the injections, patients wore sunglasses and stayed in dark rooms promptly. After a period of 36 hours, a cylindrical optical fiber with a length of 5cm (Guilin Xingda Medical Equipment Co., Ltd.) was carefully inserted and securely positioned within the tumor region for PDT using the PDT 630-IISemiconductor laser PDT instrument from Xingda^®^, China. Before photodynamic therapy, the machine needs to be calibrated. The treatment protocol involved segmented PDT, wherein the initial segment administered a power of 800mW for 10 minutes along with an energy dosage of 480J, followed by a subsequent segment delivering a power of 800mW for 5 minutes and an energy dosage of 240J. During the treatment, the doctor needs to wear specialized protective eyewear to avoid injury. From December 12th, 2022 to December 15th, 2022, a total of four PDT sessions were conducted. After the treatment, the tumor site in the colonoscopy showed extensive mucosal necrosis and shedding. The affected area exhibited obvious gray-white necrosis, allowing for smooth passage of the endoscope. Following PDT, patients underwent a 4-cycle chemotherapy regimen consisting of “Oxaliplatin + Capecitabine”, in conjunction with targeted and immunotherapy utilizing “Bevacizumab + Sintilimab”(**Figure 3**). After four cycles of sindilizumab, the patient’s thyroid function was reviewed and showed that T3 0. 83 nmol/L, T4 32. 90 nmol/L, TSH 52. 009uIU/ml, this indicated that the patient had severe hypothyroidism. The cause of hypothyroidism after immunotherapy may be due to the strong expression of PD-1/L1 in thyroid tissues, after immunotherapy, the normal thyroid cells are easily attacked by cytotoxic T-cells, which leads to hypothyroidism, so the patient needs to discontinue immunotherapy. Subsequently, the 5th cycle of chemotherapy was administered utilizing the “Oxaliplatin + Capecitabine + Bevacizumab” regimen. Following PDT, the colonoscopy re-examination conducted on March 1, 2023 revealed unobstructed lumen and significant regression of the lesion (**Supplementary Figure 1**).

After PDT, combined with chemotherapy and immunotherapy, the patient’s tumor lesions significantly regressed. The difficulty in defecation improved noticeably. Preoperative MRI showed a reduction in the degree of intestinal wall thickening. The number of enlarged lymph nodes adjacent to the bilateral iliac vessels and inguinal region decreased compared to before treatment (**Figure 1**). After Multi-Disciplinary Treatment (MDT) discussion, the decision was made to perform fluorescent laparoscopic localization combined with robotic colon cancer resection. Preoperative colonoscopy of the tumor was performed on June 6, 2023, at a distance of 15-20 cm from the anus, tumor tissue was visible with a patent lumen, and 500 ul of indocyanine green was injected into the oral and anal sides of the lesion. After perfect preparation, radical resection of malignant tumor of colon with robotic assistance was performed. The duration of the surgical procedure was 215 minutes, with intraoperative blood loss measuring 20 ml. On the first postoperative day, drainage volume reached 150 ml, and a small amount of pelvic fluid was observed during the postoperative review. No other complications were occurred, and the patient achieved ambulation on the second postoperative day, and was fed with water on the fifth postoperative day. Postoperative pathological examination of the surgical specimen showed: 1. A small amount of residual well-differentiated adenocarcinoma component in the ulcerative lesion area, with cancer invasion into the subserosal layer (pT3), without definite involvement of blood vessels or nerves; tumor regression grade (TRG): Grade 1 (single or small foci of residual cells) (**Figure 2**); no cancer invasion in the resected margins on both sides. 2. Immunohistochemical staining confirmed that there was no lymph node metastasis (0/24); immunohistochemistry staining results: cancer cells CK8/18+,Syn-, CgA-, p53 (+), C-erbB-2 (0), Ki67 (40%+); MLH1+, PMS-2+, MSH-2+, MSH-6+.

**Figure 4:**
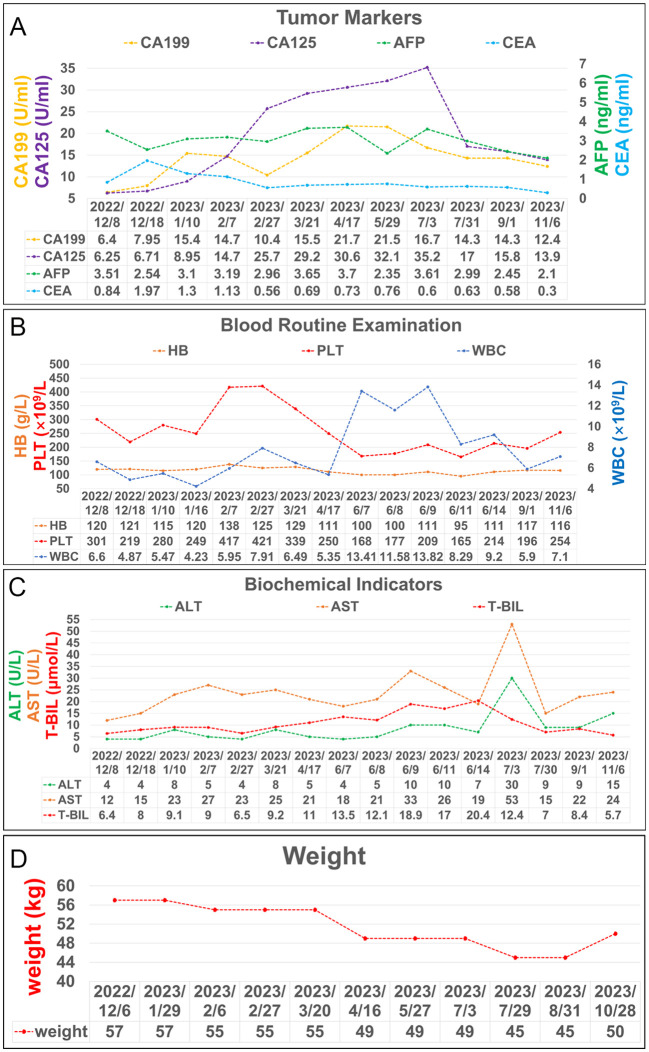
Laboratory test results during treatment. **(A)** Trends in the level of tumor markers, including AFP, CEA, CA 199 and CA125. **(B)** Trends in the level of blood routine examination, including PLT, HB, and WBC. **(C)** Trends in the level of biochemical indicators, including ALT, AST, and T-BIL. **(D)** Trends in the level of weight changes.

After undergoing regular postoperative follow-up examinations, on July 3, 2023, July 29, 2023, and August 31, 2023, the patient received chemotherapy with the regimen of “Oxaliplatin + Capecitabine + Bevacizumab”. The patient exhibited satisfactory recovery following surgery and underwent routine follow-up examinations at our institution. A CT scan of the abdomen conducted one month post-surgery revealed no significant abnormal thickening at the anastomotic site (**Figure 1**). An electronic colonoscopy demonstrated a well-formed anastomotic stoma with smooth mucosa and absence of ulcers or masses. Five months after surgery, another abdominal CT scan showed no apparent abnormal thickening at the anastomotic site. The electronic colonoscopy revealed a visible anastomotic stoma located approximately 15cm from the anus with intact mucosa and absence of ulcers or masses. The mucosa in the ascending colon appeared smooth with distinct vascular patterns and no presence of ulcers or masses was observed. Laboratory test indicators are better than before (**Figure 4**). It has been eleven months since initiation of PDT sessions and five months since robotic-assisted radical resection for malignant colonic tumor without recurrence or metastasis.

## Discussion

The majority of colon cancer patients are diagnosed at an advanced stage (16). For certain tumors, such as rectal cancer, neoadjuvant therapy has been demonstrated to effectively reduce tumor volume prior to surgery, thereby enhancing the rate of complete surgical resection and minimizing tumor recurrence and metastasis (17). Neoadjuvant therapy encompasses interventions that combine radiation therapy, chemotherapy, or immunotherapy before surgery in order to shrink the tumor volume range. This approach reduces the risk of intraoperative dissemination of tumor cells and improves the R0 resection rate during surgery. Additionally, it increases sphincter preservation rates for patients while lowering the risk of local tumor recurrence and extending disease-free survival (18, 19). Traditional neoadjuvant therapies typically involve chemotherapy, radiation therapy, immunotherapy etc (20). However, Colon tumors generally cannot be treated with radiotherapy, and there is a lack of local treatment. PDT is based on the principle of injecting a photosensitizer into the tumor tissue of patients, whereby upon exposure to light at a specific wavelength, energy transfer occurs from the photosensitizer to surrounding oxygen molecules, resulting in the generation of highly reactive singlet oxygen. Subsequently, this singlet oxygen engages in oxidation reactions with nearby biomolecules, leading to cellular toxicity and ultimately inducing apoptosis in diseased cells. By precisely targeting and eradicating affected tissues, PDT effectively combats tumors (21). PDT can serve as a local neoadjuvant treatment for colon cancer, effectively inducing tumor shrinkage within the affected area. This approach is particularly advantageous in cases of obstructive colon cancer, as it not only alleviates obstruction but also transforms emergency surgery into an elective procedure. By combining PDT with other modalities such as chemotherapy and targeted immunotherapy after relieving the obstruction, downstaging of the tumor can be achieved, ultimately enhancing surgical success rates. Although some domestic and international research teams have investigated neoadjuvant therapies for colon cancer, their focus has primarily been on evaluating the efficacy of combining capecitabine with oxaliplatin or exploring preoperative chemotherapy combined with targeted immunotherapy. Notably absent from existing literature is any investigation into incorporating PDT as part of colon cancer neoadjuvant treatment (22, 23). Further improvement is required for the efficacy of neoadjuvant chemotherapy and immunotherapy in colon cancer. A randomized controlled trial conducted by John et al (24), reported a median follow-up time of 21.1 months for patients with colon cancer who received preoperative chemotherapy alone, compared to 19.8 months for those who received chemotherapy combined with cetuximab. Another randomized controlled trial by Foxtrot et al (25), divided colon cancer patients into two groups: one group underwent preoperative chemotherapy followed by postoperative chemotherapy, while the other group only received postoperative chemotherapy. The incidence of complications did not significantly differ between the two groups (14% vs 12%, p=0.81), suggesting that preoperative chemotherapy does not decrease the occurrence of postoperative complications in colon cancer patients. Among the 99 patients who underwent preoperative chemotherapy, complete remission was achieved in only 2 cases, significant remission in another 2 cases, and minimal regression was observed in remaining 65 cases. However, in this case, after undergoing PDT combined with chemotherapy, targeted therapy, and immunotherapy, postoperative pathological examination confirmed level 1 regression with residual individual or small foci cells only; indicating successful transformation following PDT. Preoperative PDT exhibits remarkable therapeutic efficacy in the treatment of colon cancer. The inherent sensitivity of colon cancer to PDT leads to effective destruction of tumor cells (26, 27). In a study conducted by Sun et al (28) in 2016, PDT was administered to 23 patients with advanced colon cancer, while a control group consisting of 30 patients received conventional chemotherapy and radiation therapy. The results exhibited superior effectiveness of PDT compared to the control group, highlighting its significant superiority over traditional preoperative chemoradiotherapy for managing colon cancer. Moreover, complications commonly encountered in colon cancer patients such as gastrointestinal bleeding and colonic obstruction can be effectively addressed through PDT utilizing fiber optics, endoscopy, and other interventional techniques that enable direct access to the colonic lumen for prompt treatment of bleeding or alleviation of intestinal obstructions – an achievement unattainable through traditional neoadjuvant chemoradiotherapy. Traditional preoperative chemoradiotherapy inflicts substantial harm on patients and gives rise to complications; conversely, PDT exhibits minimal toxicity and fewer adverse reactions, rendering it safe and reliable for repeated treatments aimed at reducing lesion size and achieving therapeutic objectives (12). As a localized treatment modality, PDT yields comparable outcomes as neoadjuvant radiotherapy, chemotherapy or immunotherapy; thus offering an irreplaceable advantage in managing obstructed cases of colon cancer (**Supplementary Figure 2**).

Although PDT has demonstrated promising short-term efficacy as a neoadjuvant therapy for colon cancer, surgical resection remains necessary subsequent to the treatment. Xiao et al (29) discovered that employing the second-generation photosensitizer ALA for PDT on human colon cancer SW480 cells resulted in significant inhibition of cell proliferation and reduction in total viable cell count, accompanied by an increase in cell death proportion within 12-24 hours. However, this inhibitory effect was transient as the survival rate of cells began to gradually rise after 24 hours of PDT. Kashtan et al (30) conducted PDT on a patient with colorectal cancer and observed tumor necrosis and disappearance post-treatment; nevertheless, histological examination of live tissue collected at the lesion site after 16 weeks revealed the presence of cancer cells, indicating a potential risk of tumor recurrence following PDT. These research findings suggest that although PDT may exhibit favorable short-term efficacy, its long-term effectiveness remains subject to debate. Therefore, early surgery should be considered for patients who have the opportunity to undergo surgical resection subsequent to PDT treatment. In this case, it can be observed that the tumor site treated with PDT exhibited necrosis and shedding while maintaining lumen patency, indicating favorable short-term efficacy of PDT followed by curative surgery. This further underscores that early surgery following PDT can offer hope for long-term survival in patients.

There are no reports of PDT as a preoperative neoadjuvant treatment for fluorescent laparoscopic localized robotic colon cancer surgery. The integration of photodynamic neoadjuvant therapy with fluorescence-guided robotic surgery can offer more precise treatment for patients (31). For this type of colon cancer combined with obstruction, photodynamic therapy should be applied first to relieve the patient’s obstruction and relieve the symptoms, and then chemotherapy, immunotherapy, or targeted therapy should be used to reduce the size of the patient’s tumor, so as to prepare for fluorescent laparoscopic localization of the robotic surgery, and reduce the trauma, which will lead to the patient recovering faster and less likely to recur. Robotic colon cancer resection has been demonstrated to be safe and feasible. The combination of fluorescence laparoscopy and robotic surgery enables complete and accurate tumor lesion removal. Postoperative pathological results in this case exhibited absence of cancer cell invasion at both sides’ margins of the tumor, along with no observed lymph node metastasis among all 24 groups. Colonoscopy conducted two months after the surgery also revealed a smooth mucosa without any indications of tumor formation. Hence, combining photodynamic adjuvant therapy with fluorescence-guided robotic surgery may present novel therapeutic options for advanced-stage colon cancer patients while providing clinicians alternative approaches to treatment. However, it should be noted that the follow-up period in this report is relatively short, necessitating further observation to determine long-term efficacy. Additionally, extensive practice and experience accumulation involving a large number of samples remain essential.

## Data Availability

The original contributions presented in the study are included in the article/**Supplementary Material**. Further inquiries can be directed to the corresponding author.
