# Supplementary material for: Case report: A clinical report of photodynamic neoadjuvant combined with fluorescent laparoscopic localization robotic surgery for the treatment of patients with advanced colorectal cancer combined with obstruction
Source: Front Immunol. 2024 Sep 4;15:1403613. doi: 10.3389/fimmu.2024.1403613 (PMC11409080; doi:10.3389/fimmu.2024.1403613)
Supplement: Supplementary file 1 [file DataSheet1.pdf]

Table S1 Comparison of surgical treatments for colon cancer combined with obstruction[3-10]

	one-stage operation	two-stage operation	three-stage operation
operation method	One-stage tumor resection and intestinal anastomosis	One-stage resection of the primary tumor, proximal colostomy, distal closure, and two-stage stoma reduction	One-stage proximal colostomy, two-stage tumor resection, and three-stage colostomy
disadvantage	The incidence of anastomotic leakage and incision infection after one-stage resection and anastomosis was high	Low success rate of stoma recovery, easy to become permanent stoma	Long treatment period, multiple surgeries, high trauma to patients, high incidence of perioperative complications
5-year survival rate	62%	40%	39.2%
complication rate	53.8%	51.3%	23%

Table S2 Robotic versus laparoscopic surgery[13-15]

	View	Flexibility	Accuracy	Number of personnel	Operating space	Trauma
laparoscope	2D field of view with 4-5x magnification	non-rotatable	Operator-operated, less precise	High number of personnel and need to work with each other	Difficult to operate in deep abdomen and narrow space	minimally invasive
Robotic surgery	3D field of view with 10-15x magnification	540° rotatable	Filtering of physiological tremor with high accuracy	Low staffing levels, master knife control is sufficient	Deep belly and tight spaces can be advantageous	minimally invasive

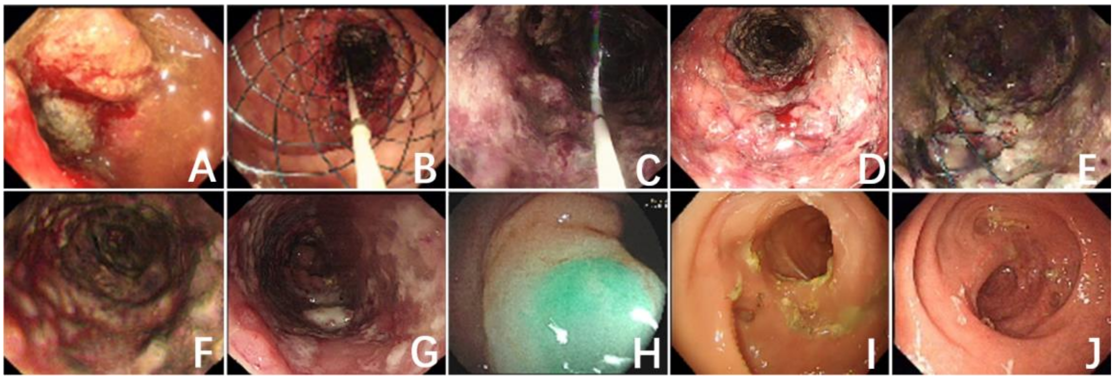

Figure S1 enteroscopy **(A)** Before photodynamic therapy. **（B）** First photodynamic therapy.**(C)** Second photodynamic therapy. **(D)** Third photodynamic therapy. **(E)** Fourth photodynamic therapy. **(F)** Four days after PDT. **(G)** Three months after PDT. **(H)** Preoperative colonoscopy. **(I)** Repeat colonoscopy 2 months after surgery. **(J)** Repeat colonoscopy 5 months after surgery.

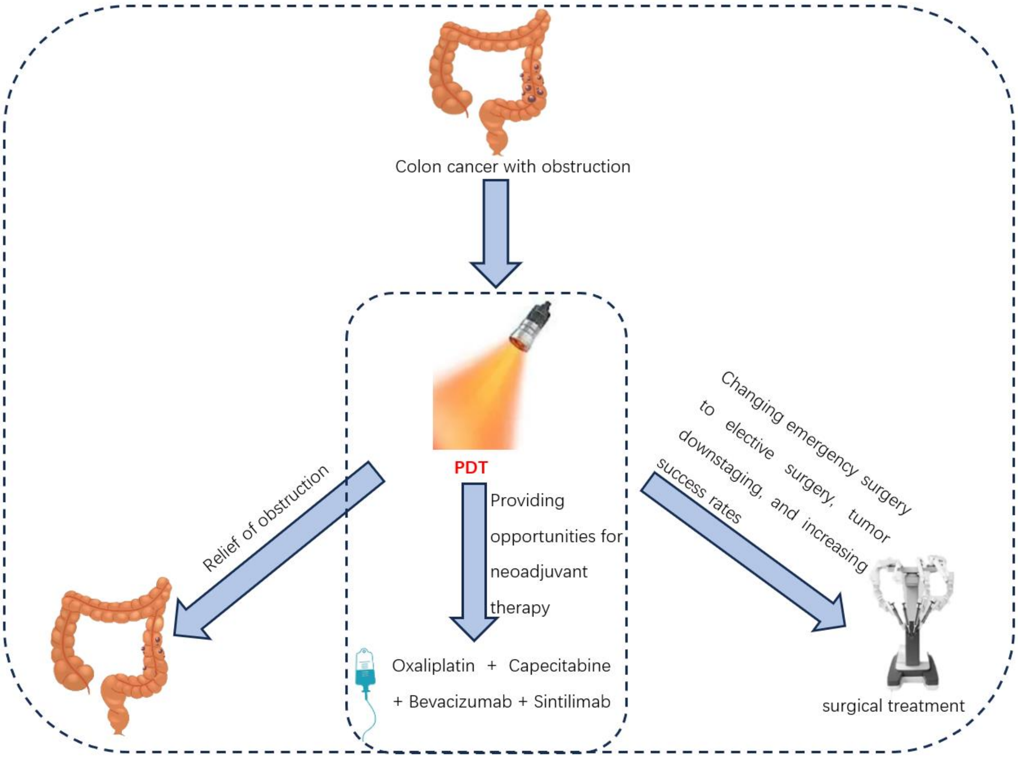

Figure S2 The central role of photodynamic therapy in combined chemotherapy, targeted, and immunotherapy for colon cancer with combined obstruction.
